# Continuous Production of Docetaxel-Loaded Nanostructured Lipid Carriers Using a Coaxial Turbulent Jet Mixer with Heating System

**DOI:** 10.3390/molecules30020279

**Published:** 2025-01-12

**Authors:** Hyeon Su Lim, Won Il Choi, Jong-Min Lim

**Affiliations:** 1 Department of Electronic Materials, Devices, and Equipment Engineering, Soonchunhyang University, 22 Soonchunhyang-ro, Shinchang-myeon, Asan-si 31538, Chungcheongnam-do, Republic of Korea; hhhsss1849@naver.com; 2 Center for Bio-Healthcare Materials, Bio-Convergence Materials R&D Division, Korea Institute of Ceramic Engineering and Technology, 202 Osongsaengmyeong 1-ro, Osong-eup, Heungdeok-gu, Cheongju 28160, Chungcheongbuk-do, Republic of Korea; 3 Department of Chemical Engineering, Soonchunhyang University, 22 Soonchunhyang-ro, Shinchang-myeon, Asan-si 31538, Chungcheongnam-do, Republic of Korea

**Keywords:** nanoparticles, nanoprecipitation, nanostructured lipid carriers, turbulent jet, mixer, microfluidics

## Abstract

The continuous synthesis of nanoparticles (NPs) has been actively studied due to its great potential to produce NPs with reproducible and controllable physicochemical properties. Here, we achieved the high throughput production of nanostructured lipid carriers (NLCs) using a coaxial turbulent jet mixer with an added heating system. This device, designed for the crossflow of precursor solution and non-solvent, combined with the heating system, efficiently dissolves solid lipids and surfactants. We reported the flow regime according to the Reynolds number (Re). Also, we confirmed the size controllability of NLCs as dependent on both Re and lipid concentration. The optimized synthesis yields NLCs around 80 nm, ideal for targeted drug delivery by enhanced permeability and retention (EPR) effect. The coaxial turbulent jet mixer enables effective mixing, producing uniform size distribution of NLCs. The NLCs prepared using the coaxial turbulent jet mixer were smaller, more uniform, and had higher drug loading compared to the NLCs synthesized by a bulk nanoprecipitation method, showcasing its potential for advancing nanomedicine.

## 1. Introduction

During the previous decades, the number of research papers reporting nanostructured lipid carrier (NLC)-based formulations has significantly increased. The rise in the NLC research is intrinsically attributed to various advantages including high drug loading, long-term drug stability, biocompatibility, biodegradability, ability to penetrate the blood–brain barrier (BBB), etc. [1,2,3,4,5] In most cases, the preparation of NLCs is conducted by a conventional bulk synthesis method under elevated temperatures to enhance the solubility of lipids and surfactants [4]. The conventional bulk synthesis for nanoparticles (NPs) is inherently discontinuous and suffers from the limited controllability and reproducibility of physicochemical properties [6,7,8]. These limitations impede scalable production and consistent quality, posing challenges for regulatory approval and clinical applications. To address these shortcomings, continuous microfluidic techniques have emerged as a potential solution. However, despite their promising advantages in improving controllability and reproducibility, microfluidic methods still face challenges in achieving mass production capabilities [9]. This bottleneck restricts their utility for clinical and industrial applications. The coaxial turbulent jet mixer has recently emerged as an innovative platform, offering controllability and reproducibility while enabling the large-scale continuous production of various nanoparticles including polymeric NPs, iron oxide NPs, and liposomes at ambient temperatures [5,10,11,12].

In parallel, docetaxel (DTX) has received significant attention for its incorporation into nanoparticle-based drug delivery systems due to its poor water solubility and systemic toxicity in free-drug form. Recent clinical research has highlighted the potential of DTX-containing nanoparticles in improving drug delivery efficiency, reducing side effects, and enhancing therapeutic outcomes. For example, DTX-loaded polymeric nanoparticles, such as BIND-014, have demonstrated improved pharmacokinetics and antitumor efficacy in early-phase clinical trials compared to traditional formulations [13,14]. These advances highlight the clinical relevance of developing scalable, reproducible, and efficient synthesis methods for DTX-loaded nanoparticles. However, to our best knowledge, no experimental study has been reported to synthesize NLCs by creating high-temperature environments in high throughput continuous production with the flash nanoprecipitation method.

In this study, we successfully expanded the capability of the coaxial turbulent jet mixer by incorporating a heating system, thereby achieving the high-temperature synthesis of NLCs while preserving the advantages of homogeneity, controllability, and reproducibility. The integration of a heating system involving hot plates adjacent to each pump minimizes temperature drops within the pumps, dampers, and tube fittings, thus elevating the temperature of both the surroundings and the device itself. As a result, NLC synthesis was achieved without the need for a separate temperature-controlled room. The effects of lipid concentration and Reynolds number (Re) were systematically changed to control the average NPs size and the polydispersity index (PDI). The optimized DTX-loaded NLC formulation with uniform size distribution was characterized for drug loading, encapsulation efficiency, and release profile. The DTX-loaded NLCs synthesized with the coaxial turbulent jet mixer exhibited higher drug loading and encapsulation efficiencies, ensuring improved therapeutic efficacy. Release profile analyses indicated comparable delayed release behavior between the DTX-loaded NLCs produced using the coaxial turbulent jet mixer and those from bulk methods, even though the DTX-loaded NLCs prepared using a coaxial turbulent jet mixer are significantly smaller. The combination of the coaxial turbulent jet mixer and the integrated heating system has enabled the large-scale and continuous production of NLCs with controlled size distribution and enhanced drug loading characteristics, which are crucial for evaluating their potential efficacy and stability for drug delivery applications.

## 2. Results and Discussion

### 2.1. Coaxial Turbulent Jet Mixer for Continuous Synthesis of Nanostructured Lipid Carriers

**Scheme 1:**
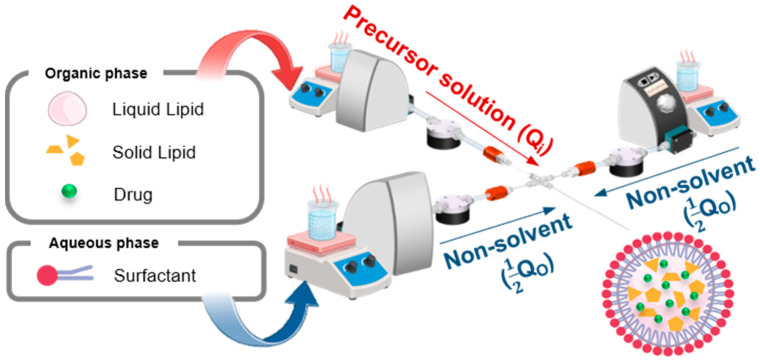
Coaxial turbulent jet mixer with heating system. The organic phase containing liquid lipid, solid lipid, and drug flows through the inner stream. The aqueous phase containing surfactant flows through the outer stream. Both the organic and aqueous phases are heated to 60 °C.

The coaxial turbulent jet mixer was specifically engineered to enable the effective intersection of a precursor solution and a non-solvent in a crossflow configuration. Structurally, the precursor solution occupies the inner circle of the concentric flows, while the non-solvent surrounds it in the outer circle. To optimize the dissolution efficiency of solid lipids and surfactants, we incorporated a heating system into the mixer (Scheme 1). The heating configuration included strategically positioned hot plates adjacent to each pump for preheating the solutions. Additionally, electric heaters and hot packs ensured consistently elevated temperatures within the pumps, dampers, and tube fittings, effectively minimizing temperature gradients. This design innovation eliminated the need for a dedicated hot room, simplifying the synthesis process while maintaining precise temperature control. The resulting system demonstrated high versatility and efficiency in synthesizing nanostructured lipid carriers (NLCs).

### 2.2. Flow Regime Characterization by Reynolds Number

**Figure 1:**
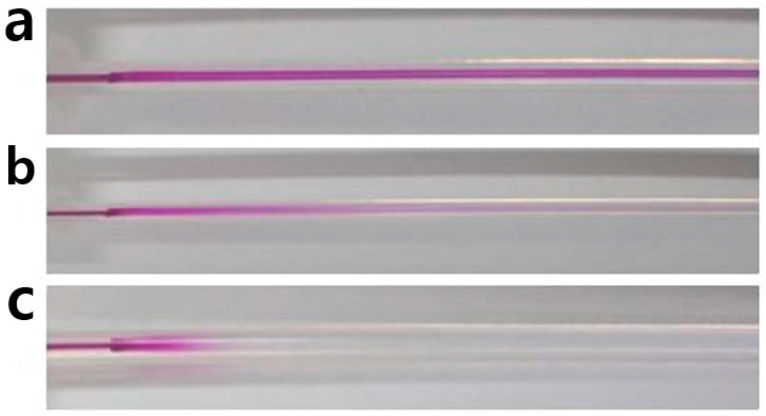
The top views of fluid flow in the coaxial turbulent jet mixer when the flow is (**a**) laminar flow (Re = 423.0), (**b**) transitional flow (Re = 846.1), and (**c**) turbulent jet flow (Re = 2115.2), respectively. Here, the flow rate ratio (R) is fixed at 0.067.

To further validate the role of the Re, we explored its influence on the critical mixing properties of the coaxial turbulent jet mixer. To characterize flow regimes, we visualized fluid flow patterns as a function of the Re (Figure 1). Here, the flow rate ratios (R = Q_i_/Q_o_) were maintained at R = 0.067. At Re = 423.0, the inner stream, represented by a purple dye, exhibited laminar flow with minimal mixing downstream (Figure 1a). At Re = 846.1, the flow transitioned, showing partial mixing that became complete further downstream (Figure 1b). At Re = 2115.2, the flow achieved a fully turbulent jet, resulting in rapid and homogeneous mixing (Figure 1c). The measurements of mixing time and energy dissipation provided quantitative insights into how turbulence intensity directly correlates with particle uniformity. For instance, at Re = 2115.2, the rapid mixing facilitated by the fully developed turbulent flow created a homogeneous reaction environment for the flash nanoprecipitation of NLCs. Achieving uniformly sized NLCs during the flash nanoprecipitation requires a turbulent jet flow at the intersection of the precursor solution and non-solvent. The mixing time (τ_mix_) was significantly reduced under turbulent conditions compared to transitional flow, highlighting the importance of Re in controlling the mixing efficiency. Higher Re can be achieved by increasing the overall flow rate using additional pumps, enabling shorter mixing times to expedite the mixing process.

### 2.3. Tunable Size Control of NLCs

Nanoparticles (NPs) in the size range of 20–200 nm are optimal for leveraging enhanced permeability and retention (EPR) effects in solid tumor treatment while avoiding filtration by the liver and spleen [15,16]. However, the EPR effect is dependent on cancer type and NP characteristics, with recent studies indicating its efficacy for NPs in the range of a few nanometers to 100 nm [17]. Our target was to produce NLCs with a mean size of approximately 80 nm while maintaining tunability for diverse applications.

**Figure 2:**
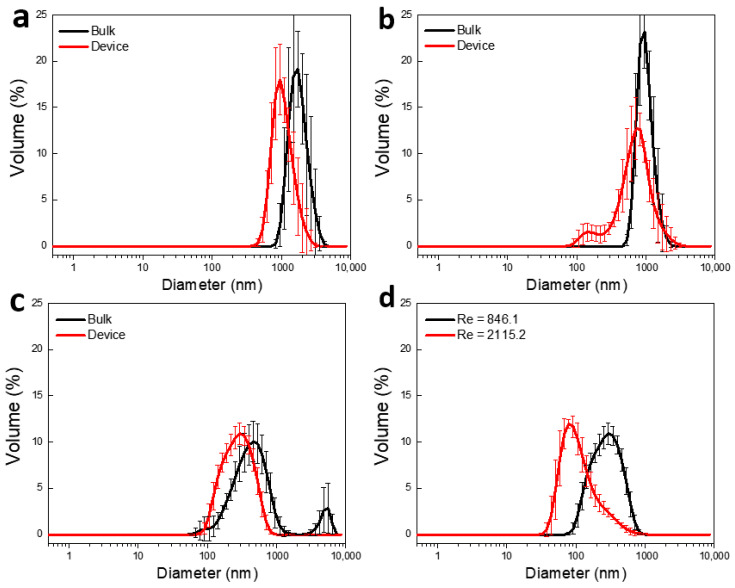
(**a**–**c**) Size distribution of NLCs without DTX fabricated by bulk synthesis and coaxial turbulent jet mixer (Re = 846.1). Here, the lipid concentrations in the organic phase are (**a**) 5% (*w*/*v*), (**b**) 3% (*w*/*v*), and (**c**) 1% (*w*/*v*), respectively. (**d**) Size distribution of NLCs fabricated by using coaxial turbulent jet mixer when Re = 846.1 and Re = 2115.2. Here, lipid concentration in the organic phase is fixed at 1% (*w*/*v*).

**Figure 3:**
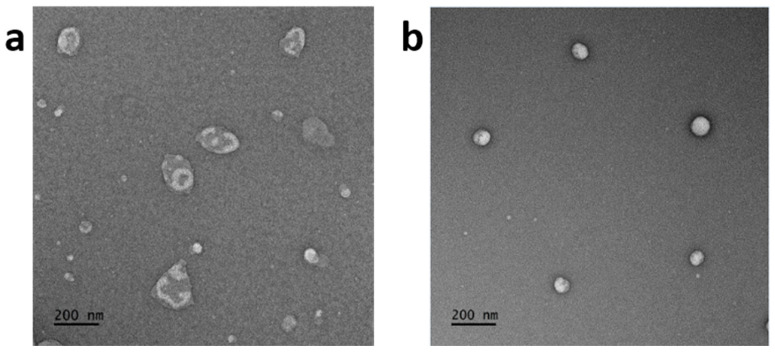
TEM images of the NLCs without DTX fabricated using (**a**) bulk synthesis and (**b**) the coaxial turbulent jet mixer (Re = 2115.2).

Dynamic light scattering (DLS) analyses revealed that the NLCs synthesized using the coaxial turbulent jet mixer were consistently smaller than those produced by the bulk synthesis method. At Re = 846.1, reducing lipid concentration in the organic solvent from 5% (*w*/*v*) to 1% (*w*/*v*) resulted in a significant decrease in particle size (Figure 2a–c). For a 1% lipid concentration, the average particle size was approximately 300 nm. Increasing the Re to Re = 2115.2 under identical lipid concentrations further reduced the average particle size to 80 nm (Figure 2d). These findings were corroborated by transmission electron microscopy (TEM), where size distributions from TEM images (Figure 3a,b) aligned with DLS measurements. This capability for fine-tuned size control underscores the versatility of the coaxial turbulent jet mixer for producing application-specific NLCs.

### 2.4. Drug Loading and Release Characteristics

**Figure 4:**
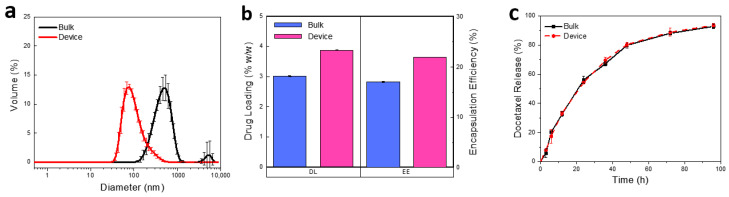
DTX-loaded NLCs prepared by bulk synthesis and the coaxial turbulent jet mixer (Re = 2115.2). (**a**) Size distribution, (**b**) drug loading and encapsulation efficiency, and (**c**) release profile of DTX from the NLCs, respectively.

Lipid-based nanoparticles are pivotal in nanomedicine due to their ability to deliver hydrophobic drugs effectively [18,19,20,21]. DTX, a model chemotherapeutic agent, was encapsulated in NLCs by incorporating it into the precursor solution prior to nanoprecipitation. The resulting DTX-loaded NLCs prepared using the coaxial turbulent jet mixer exhibited an average size of ~80 nm, comparable to empty NLCs, without significant size variation (Figure 4a). At Re = 2115.2, the production rate of DTX-loaded NLCs was 288 mg/min when the lipid concentration in the organic solvent was 1% (*w*/*v*). This production rate is relevant to 151 kg/year, sufficient to generate enough material for Phase-I clinical studies [14]. In contrast, bulk-synthesized NLCs showed a larger particle population, with main peaks near 500 nm. Drug loading and encapsulation efficiencies were notably higher in the NLCs produced by the coaxial turbulent jet mixer, as shown in Figure 4b. Release profile analyses demonstrated that the half-life of DTX in NLCs was approximately 20 h, with sustained release over time (Figure 4c). The release profiles are similar between the DTX-loaded NLCs synthesized through bulk synthesis and using the coaxial turbulent jet mixer even though the DTX-loaded NLCs prepared using the coaxial turbulent jet mixer are significantly smaller. This underscores the advantages of the coaxial turbulent jet mixer in producing homogeneous, high-quality NLCs with efficient drug loading and controlled release characteristics.

## 3. Materials and Methods

### 3.1. Materials

Compritol^®^ 888 ATO was purchased from Gattefossée (Saint-Priest, France). Oleic acid, phenolphthalein, ethanol, and Kolliphor^®^ P 407 were purchased from Sigma-Aldrich (St. Louis, MO, USA). Tetrahydrofuran (THF) was purchased from Honeywell (Charlotte, NC, USA). Sodium hydroxide (0.1 N) was purchased from Burdick & Jackson (Muskegon, MI, USA). Hydrogen chloride (0.1 N) was obtained from Daejung Chemicals (Siheung-si, Republic of Korea). DTX was purchased from LC Laboratories (Woburn, MA, USA). For imaging purposes, uranyl acetate was obtained from Electron Microscopy Sciences (Hatfield, PA, USA).

### 3.2. Fabrication of Coaxial Turbulent Jet Mixer

The coaxial turbulent jet mixer was fabricated according to the methods detailed in our previous studies [22,23]. Polytetrafluoroethylene (PTFE) tubes (KNF Flodos AG, Sursee, Switzerland) with an outer diameter (OD) of 6 mm and an inner diameter (ID) of 4 mm were connected to diaphragm pumps and pulsation dampers using polyethylene (PE) ferrules and nuts (KNF Neuberger GmbH, Freiburg im Breisgau, Germany). A fluorinated ethylene propylene (FEP) tube (Upchurch Scientific, Oak Harbor, WA, USA) with an OD of 1 mm and an ID of 0.5 mm was used for the inner stream. Three FEP tubes (Upchurch Scientific) with an OD of 3.175 mm and an ID of 1.55 mm were used for two outer stream tubes and one outlet stream tube. The transition fittings (BOHLENDER GmbH, Grünsfeld, Germany) were employed to facilitate the transitions in diameter between different types of tubes. The inlet tubes for an inner stream and two outer streams were connected to an ethylene tetrafluoroethylene (ETFE) cross-body tube fitting (Upchurch Scientific) using ETFE ferrules and nuts. A FEP sleeve (Upchurch Scientific) with an OD of 1.59 mm and an ID of 1.07 mm was used to secure the thin FEP tube for the inner stream within the cross-body fitting, allowing it to penetrate and align coaxially with an outlet FEP tube with an OD of 3.175 mm and an ID of 1.55 mm.

### 3.3. Characterization of Fluid Flow in the Coaxial Turbulent Jet Mixer

The characterization of fluid flow within the coaxial turbulent jet mixer was conducted following the experimental procedures from our previous studies [10,11,22,23]. Phenolphthalein (Sigma-Aldrich) was used to visualize the mixing dynamics. Because phenolphthalein is insoluble in water, a solution of 0.1 N sodium hydroxide (Burdick & Jackson) in a water–ethanol mixture (1:2 volume ratio) containing 1% (*w*/*v*) phenolphthalein served as the pink-colored basic inner solution. The outer solution consisted of 0.1 N hydrogen chloride (Daejung Chemicals) in a water–ethanol mixture (1:2 volume ratio). To ensure that the densities of both the inner and outer fluids matched, the same water–ethanol mixture composition was used in preparing both the basic inner solution and the acidic outer solution. Complete mixing was indicated by the phenolphthalein transitioning from pink to colorless, which indicated that the mixed solution became neutral or acidic. The average Re in the outlet tube was calculated using Re = QD/νA, where Q is the total flow rate, ν is the kinematic viscosity, D is the outlet tube diameter, and A is the cross-sectional area. The ν value for the water–ethanol mixture was determined using the reported values of dynamic viscosity (μ) and density (ρ) for the water–ethanol mixture [24]. Re could be controlled by adjusting the total flow rates, assuming constant mixer geometry and solution composition.

### 3.4. Preparation of Docetaxel-Loaded Nanostructured Lipid Carriers Using Bulk Synthesis Method

Compritol^®^ 888 ATO and oleic acid (3:1 *w*/*w*) were dissolved in a THF-ethanol solvent mixture (3:1 *v*/*v*) at concentrations of 1%, 3%, and 5% (*w*/*v*) at 60 °C. For drug encapsulation, DTX was added to the precursor solution at 15 wt% of the total lipid weight. A 1% (*w*/*v*) Kolliphor^®^ P 407 aqueous solution served as the non-solvent phase. The non-solvent phase was also heated to 60 °C to prevent the heat transfer between the precursor solution and the non-solvent phase. For bulk synthesis, a modified solvent injection method was used with a precursor solution to non-solvent volume ratio of 1:15 [25]. The precursor solution was rapidly injected into the non-solvent under magnetic stirring for three hours at room temperature. The synthesized NLC suspensions were purified by ultrafiltration at 3000 rpm for 8 min using Amicon Ultracel 100 K membrane filters (Sigma-Aldrich) and re-dispersed in deionized water three times.

### 3.5. Preparation of Docetaxel-Loaded Nanostructured Lipid Carriers Using Coaxial Turbulent Jet Mixer with Heating System

In the flash nanoprecipitation method using the coaxial turbulent jet mixer, the precursor solution and non-solvent were the inner and outer streams, respectively. As the precursor solution, Compritol^®^ 888 ATO and oleic acid were prepared as described in the bulk synthesis method. The Re and kinematic viscosity (ν) were estimated using the total flow rates (Q) controlled by diaphragm pumps with dampers and the previously reported values of dynamic viscosity (μ) and density (ρ) in water-THF mixtures [26]. The coaxial turbulent jet mixer was equipped with a heating system, ensuring that both the precursor solution and non-solvent streams were maintained at 60 °C during the flash nanoprecipitation. The synthesized NLC suspensions were purified in the same manner as those prepared by the bulk synthesis method.

### 3.6. Characterization of Nanostructured Lipid Carriers

The size distributions by volume percent of the synthesized NLCs were measured using dynamic light scattering (DLS) with a Zetasizer Nano ZS (Malvern Instruments Ltd., Malvern, UK). The measurements were performed in triplicate under identical conditions, and the average size distributions were calculated and plotted for reproducibility and accuracy. For morphological analysis, the synthesized NLCs were stained with uranyl acetate (Electron Microscopy Sciences) to enhance contrast and imaged using transmission electron microscopy (TEM, JEOL JEM-1400 plus, Tokyo, Japan). The encapsulation efficiency and loading capacity of DTX in the NLCs were quantified using high-performance liquid chromatography (HPLC, Shimadzu 20A system, Kyoto, Japan) following precise calibration and validation steps to ensure the accurate measurement of DTX concentration [27,28]. These comprehensive characterization methods provided detailed insights into the physical and chemical properties of the NLCs, including their size, morphology, and drug loading efficiency, which are crucial for evaluating their potential efficacy and stability for drug delivery applications.

## 4. Conclusions

The integration of a heating system into the coaxial turbulent jet mixer significantly enhances the large-scale and continuous synthesis of lipid-based nanoparticles by facilitating the efficient dissolution of solid lipids and surfactants. This system includes strategically placed hot plates adjacent to each pump, which effectively minimize temperature drops within the pumps, dampers, and tube fittings, ensuring consistent elevated temperatures throughout the system. This innovation eliminates the need for a separate controlled environment, simplifying the synthesis process while maintaining efficiency. The study also investigated the effects of varying Re on the flow regime within the coaxial turbulent jet mixer, a critical factor for achieving uniform nanoparticle precipitation through rapid mixing. The visualization of fluid flow revealed distinct flow patterns corresponding to different Re, demonstrating the transition from laminar to turbulent flow as the Re increased. The results showed that at Re = 2115.2, the coaxial turbulent jet mixer achieved effective turbulent mixing, essential for synthesizing NLCs with uniform size distribution. In terms of size control, the coaxial turbulent jet mixer consistently produced NLCs with smaller diameters compared to bulk synthesis methods. By optimizing parameters such as lipid concentration and Re, the system successfully synthesized NLCs with an average size of approximately 80 nm, closely matching the target size for the EPR effect. These results underscore the precision and flexibility of the coaxial turbulent jet mixer in achieving application-specific size requirements. At Re = 2115.2, the production rate of DTX-loaded NLCs were 288 mg/min when the lipid concentration in the organic solvent was 1% (*w*/*v*). This production rate is relevant to 151 kg/year, sufficient to generate enough material for Phase-I clinical studies. The scalability of this method further indicates its potential for industrial process chemistry production lines, making it a promising solution for high-demand applications in nanomedicine. In addition, the DTX-loaded NLCs synthesized with the coaxial turbulent jet mixer exhibited higher drug loading and encapsulation efficiencies, ensuring improved therapeutic efficacy. Release profile analyses indicated comparable delayed release behavior between the DTX-loaded NLCs produced using the coaxial turbulent jet mixer and those from bulk methods, even though the DTX-loaded NLCs prepared using the coaxial turbulent jet mixer are significantly smaller. The combination of the coaxial turbulent jet mixer and the integrated heating system has enabled the large-scale and continuous production of NLCs with controlled size distribution and enhanced drug loading characteristics.

## Data Availability

Data are contained within the article.
